# How experiences become data: the process of eliciting adverse event, medical history and concomitant medication reports in antimalarial and antiretroviral interaction trials

**DOI:** 10.1186/1471-2288-13-140

**Published:** 2013-11-14

**Authors:** Elizabeth N Allen, Adiel K Mushi, Isolide S Massawe, Lasse S Vestergaard, Martha Lemnge, Sarah G Staedke, Ushma Mehta, Karen I Barnes, Clare IR Chandler

**Affiliations:** 1 Division of Clinical Pharmacology, Department of Medicine, University of Cape Town, Cape Town, South Africa; 2 National Institute for Medical Research, Dar es Salaam, Tanzania; 3 National Institute for Medical Research, Tanga Centre, Tanga, Tanzania; 4 Centre for Medical Parasitology at Institute of International Health, Immunology and Microbiology, University of Copenhagen, Copenhagen, Denmark; 5 Department of Infectious Diseases, Copenhagen University Hospital, Copenhagen, Denmark; 6 Department of Clinical Research, London School of Hygiene & Tropical Medicine, Bloomsbury, London, UK; 7 Department of Global Health & Development, London School of Hygiene & Tropical Medicine, Bloomsbury, London, UK

**Keywords:** Clinical trial, Safety, Harm, Pharmacovigilance, Malaria, HIV, Elicitation, Social context, South Africa, Tanzania

## Abstract

**Background:**

Accurately characterizing a drug’s safety profile is essential. Trial harm and tolerability assessments rely, in part, on participants’ reports of medical histories, adverse events (AEs), and concomitant medications. Optimal methods for questioning participants are unclear, but different methods giving different results can undermine meta-analyses. This study compared methods for eliciting such data and explored reasons for dissimilar participant responses.

**Methods:**

Participants from open-label antimalarial and antiretroviral interaction trials in two distinct sites (South Africa, n = 18 [all HIV positive]; Tanzania, n = 80 [86% HIV positive]) were asked about ill health and treatment use by sequential use of (1) general enquiries without reference to particular conditions, body systems or treatments, (2) checklists of potential health issues and treatments, (3) in-depth interviews. Participants’ experiences of illness and treatment and their reporting behaviour were explored qualitatively, as were trial clinicians’ experiences with obtaining participant reports. Outcomes were the number and nature of data by questioning method, themes from qualitative analyses and a theoretical interpretation of participants’ experiences.

**Results:**

There was an overall cumulative increase in the number of reports from general enquiry through checklists to in-depth interview; in South Africa, an additional 12 medical histories, 21 AEs and 27 medications; in Tanzania an additional 260 medical histories, 1 AE and 11 medications. Checklists and interviews facilitated recognition of health issues and treatments, and consideration of what to report. Information was sometimes not reported because participants forgot, it was considered irrelevant or insignificant, or they feared reporting. Some medicine names were not known and answers to questions were considered inferior to blood tests for detecting ill health. South African inpatient volunteers exhibited a “trial citizenship”, working to achieve researchers’ goals, while Tanzanian outpatients sometimes deferred responsibility for identifying items to report to trial clinicians.

**Conclusions:**

Questioning methods and trial contexts influence the detection of adverse events, medical histories and concomitant medications. There should be further methodological work to investigate these influences and find appropriate questioning methods.

## Background

Assessments of drug harm and tolerability rely, in part, on clinical trial participant reports of adverse events (AEs), medical histories and concomitant medications. However, there is no consensus regarding the detail of how such reports should be elicited, in particular how participants should be questioned about ill health and their use of medications other than the study drug(s). Heterogeneity in elicitation methods provides potential for measurement error if questioning methods are sub-optimal, and undermines meta-analyses of adverse effects [1,2]. Staff may use general enquiries to identify AEs, such as 'How have you been feeling?’, yet the impact of variation in phraseology between or within trials is unclear [3]. Some trials elicit responses using detailed, structured approaches like symptom lists, often aimed at ascertaining anticipated adverse effects or AEs of special interest, though detection of unanticipated effects may remain unsystematic [4,5]. Questioning may also involve self-completed forms or diaries. There is evidence that more detailed elicitation techniques increase the sensitivity of participant-reported AEs [6]. However, their effect on the nature of reports is unclear. Barber and Santanello [7] found that only what patients considered more bothersome was reported spontaneously when compared to use of a checklist of possible events. This suggests that spontaneously reported AEs may be more clinically meaningful, and supports concerns that detailed methods produce 'noise’: clinically irrelevant AEs that cannot be distinguished from background rates [6,8] Wernicke et al. [9] have also proposed that spontaneous reporting provided larger drug-placebo differences more often than solicitation. There is a dearth of research about methods for the elicitation of previous or concomitant medications in trials, despite evidence that participants fail to report use of other antimalarials when asked [10].

A major challenge in determining the best way to elicit these data is the lack of a gold standard to assess validity of responses; an absolute measure of what patients experience is likely unachievable. However, in other areas of pharmacoepidemiology, including case–control or cohort studies and administrative databases, there has been methodological investigation regarding the accuracy of self-reported past medical conditions and treatments, through comparison with medical or prescription records [11]. In those contexts, recall of medical history appears dependent on the type of condition, its perceived importance and a willingness to share information. Pattern of use is influential in recall of past medications, and indication- or medication-specific questions increase prevalence estimates compared to open-ended questions [12].

Factors that shape reporting of beneficial or adverse effects of pharmacotherapy is a poorly researched area [13], although a variety of relevant factors have been proposed in different research areas. These include cultural variations in how health issues are perceived [14]; negative emotions [15]; beliefs about the medication [16]; response-shift, whereby ill health is not viewed as such anymore [17]; and gender or language differences that impede communication between patients and health workers [18-20]. Belief that drugs must cause certain [side] effects as part of the therapeutic process may also play a role [21]. Specifically within the trial context it has been proposed that there may be fear of lack of confidentiality, stigma or negative repercussions as a result of reporting AEs [3,22]. Nocebo effects (harmful response to an inactive product) may be related to information about anticipated effects given to participants during the consent process [23,24].

This study aimed to identify factors shaping reports of medical histories, AEs and previous or concomitant medications by participants who had malaria and/or HIV and were enrolled in trials of concomitant antiretroviral (ARV) and antimalarial treatments. This is to inform elicitation practices for improving reporting in these clinical contexts. The ACT Consortium [25] is a global research partnership aiming to answer key questions on the delivery of artemisinin-based combination therapy (ACT) for the treatment of malaria. The Consortium involves a number of projects that elicit safety data from trial participants, providing an opportunity to investigate factors influencing data in areas where, due to high rates of illiteracy, it would be challenging to use self-completed questionnaires. This methodological study was nested in two open-label antimalarial and antiretroviral interaction trials (referred to as parent trials hereafter) that both aimed to assess the safety and blood drug concentrations (pharmacokinetics) of the widely-used antimalarial drug, artemether-lumefantrine (AL). The two different study designs and contexts provided an opportunity to explore the influence of a variety of factors on reporting. A small South African trial investigated the use of AL in HIV-infected volunteers, while a larger Tanzanian trial investigated AL in patients infected with malaria and/or HIV.

## Methods

### Study design and objectives

This was a mixed method study. Parent trial participants were asked about ill health and treatment use (in order to record AEs, concomitant medicines and medical history) through a variety of elicitation methods: general enquiries, checklists, in-depth interviews and focus group discussions. The primary objectives were to explore qualitatively participants’ experiences of illness and treatment, their reporting behaviours, and their responses to different questioning methods. Trial clinicians’ experiences with eliciting and recording participant-reported data were also explored for their perspective on these interactions. Secondly, the study aimed to compare the number and nature of data obtained through use of different question methods. It was assumed that the parent trials should seek medical history data sufficient to determine eligibility and inform the causality assessment of AEs. In addition, they should detect changes in health or treatment use from baseline such that within-trial or pooled statistical analyses could establish signals for, or evidence of harm or poor tolerability, or robust evidence for the absence of harm within the study population.

### Study population

The study population was participants and trial clinicians involved with the 2 ACT Consortium parent trials. In South Africa, this was 18 trial participants between November 2009 and February 2010, in Tanzania, 80 of 832 trial participants between October 2010 and May 2011.

Both trials investigated responses to AL, although with different designs and participant populations. The South African site was an urban tertiary hospital serving a socio-economically diverse population. In this pharmacokinetic and safety trial, groups of otherwise healthy HIV positive volunteers were recruited and admitted for 4 days to a pharmacology research ward. Prior to this, participants had been followed-up after a single dose of AL. At the time of this nested study they were returning for a multiple-dosing period of AL twice daily for 3 days, which involved intensive pharmacokinetic sampling (to determine drug levels of AL), plus follow-up on an outpatient basis until Day 21. The Tanzanian site was a rural district hospital serving a predominantly very poor population. In this efficacy, safety and pharmacokinetic trial, HIV positive or negative patients presenting with malaria symptoms were recruited from routine clinics and attended trial consultations individually as out-patients until Day 42. Those positive for malaria received the same 6-dose AL regimen as for South Africa, while those testing negative were managed according to their alternative diagnosis.

In both sites, participants who reported additional information in response to checklist questioning compared with a general enquiry were eligible to participate in in-depth interviews and subsequent focus group discussion. This was to explore their disparate responses to different questioning methods. Trial clinicians from both sites were also invited to a focus group discussion once the nested study was complete.

### Elicitation methods

Three elicitation methods were compared sequentially: general enquiry, check list and in-depth interview. The general and checklist enquires were conducted on the same day at the baseline and a follow-up visit; the in-depth interviews were conducted within a week of the follow-up visit.

#### *General enquiry*

General open-ended questions (without reference to particular conditions, body system or medications) about health and treatment use at baseline and follow-up visits were routine in both trials. At the first visit, medical and treatment histories were obtained using open ended questions. However, as the South African participants were returning for a second dosing period, these were recorded in the trial files as AEs and concomitant medications, unless they pre-dated the parent trial. Responses were probed according to common clinical practice in eliciting a medical and treatment history, which is based on the opinion of the enquirer regarding what further details would be required.

#### *Checklists*

At the first visit and a follow-up visit (3 to 7 days post enrolment), the general enquiry was immediately followed with a checklist of questions asked by the trials’ staff focusing on body systems, symptoms, diseases and treatments. A majority of fields in the checklists were common to both trials (Additional file 1) although they could not be harmonised fully. This should not have affected this study, with its primary focus on the experience of differential reporting rather than validity of the question methods. Data reported for the first time in response to checklists were probed as for the general enquiry, and recorded in separate fields so that it was clear which question method detected which data.

#### *In-depth interviews*

Of those participants who reported additional information in response to the checklist compared to the general enquiry, a convenience sample was invited to an in-depth interview to explore their disparate responses (described below). These interviews also provided an opportunity to elicit further relevant trial data using a prompted narrative of the participant’s trial experience, reflection on previous ill health and treatments, and photographs of typical over-the-counter and traditional medicines available to the study populations. These data could then be compared with data from the other elicitation methods.

### Evaluation of reporting behaviours and elicitation methods in context

Qualitative experiences of trial participants were explored through in-depth interviews and focus group discussions.

#### *In-depth interviews*

Twenty-seven participants who reported additional information in response to the checklist elicitation method compared to general enquiries were invited to participate in an in-depth interview. The informed consent document explained that the interviewer would talk about participants’ experiences of their health and use of treatments but not that the interviews were specifically looking to explore differences in reporting. The interviews lasted one to two hours and were conducted away from the trial venue by a local qualitative researcher who understood concepts relating to collecting trial safety data. After attempting to discover further relevant data using the narrative method described above, participants were asked for help in exploring why their reporting differed by question method, with reference to their own trial data and reports.

#### *Focus group discussions*

The same participants who had participated in the in-depth interviews were invited to participate in a focus group discussion within a month of completing the parent trial. The rationale for using focus group discussions was the expectation that further information about the same concepts may be revealed after reflection in a group context. In addition, unlike the in-depth interviews, information from focus group discussions could not influence their ongoing trial participation, therefore participants may have felt more comfortable talking about particular information that they had chosen not to report during the trial. Two focus group discussions were held in each country. For each (with four to eight participants, separated by HIV status to encourage openness) the facilitator explored treatment use, the meaning or importance of AEs, barriers to reporting and recommendations for improving accuracy and completeness of clinical trial data.

One focus group discussion was also held with the trial clinicians from both parent trials in March 2011. Topics included the appropriate level of data elicitation needed for adequate assessment of harm and tolerability, the relative merits of questioning methods, hypothesised barriers to accurate reporting, and suggestions for improvement.

Question guides for the participant in-depth interviews and focus group discussions were piloted and developed iteratively as data emerged. Tanzanian question guides were translated into Kiswahili using a forward-backward translation from English. EA led South African discussions in English, assisted by English-isiXhosa or Shona speaking social scientists depending on participants’ home languages. Tanzanian discussions with trial participants were conducted in Kiswahili by AM and IM. EA and AM conducted the focus group discussion with trial clinicians in English.

### Data management and analysis

AEs, medical histories and medications elicited from participants in the sampling frame, including additional reports from those who attended in-depth interview, were described statistically by elicitation method. The nature of reports was considered in relation to trial eligibility for medical and concomitant medication histories, and severity for AEs. Audio recordings of the interviews and focus group discussions were transcribed in the original language (English, isi-Xhosa or Kiswahili), translated into English where necessary, checked for quality and imported into NVivo 8 (QSR International, 2009) with summaries and observations written by interviewers directly after each interview. Transcripts were analysed thematically in terms of explanations given for differential reporting by question method, and how participants expressed themselves. Relevant text was examined by EA for repeating ideas, which were labeled and grouped into themes reflecting the underlying meaning or concepts behind statements [26,27]. The emerging coding structure was revised after each transcript, with on-going review by CC. EA and CC explored the emerging themes in relation to broader theories.

### Ethical considerations

Approval was obtained from the ethics committees of the University of Cape Town, Faculty of Health Sciences and the Tanzanian National Institute of Medical Research Coordinating Committee. Trial participants were told who would have access to their responses and that refusal to participate was without disadvantage for subsequent care or place in the parent trial. Informed consent processes and forms were available in English and local languages. Transport reimbursement and refreshments were provided at interviews and focus group discussions.

## Results

### Study participants

The characteristics of participants who were asked both general and checklist enquiries and the subgroup who took part in the in-depth interviews (and subsequent focus group discussions) are given in Table 1. None of the parent trial participants who were approached refused an in-depth interview; however some interviews could not be scheduled due to participant or interviewer availability.

**Table 1:** Characteristics of trial participants

		**All participants from the sampling frames**		**Subgroup of participants interviewed**	
		**South Africa (n = 18)**	**Tanzania (n = 80)**	**South Africa (n = 11)**	**Tanzania (n = 16)**
**Number (%) female**		13 (72.2)	53 (66.3)	8 (72.7)	9 (56.3)
**Median (IQR) age in years**		37.1 (33.4 - 39.7)	40 (32.0 – 36.5)	38.1 (35.0 – 42.1)	34.5 (24.5 – 48.5)
**Number (%) HIV positive**		18 (100.0)	69 (86.3)	11 (100.0)	10 (62.5)
**Number taking ARVs**		18 (100.0)	63 (78.8)	11 (100.0)	9 (56.3)
**Highest education completed number (%)**		Data not available for South Africa		Unknown for 1 participant in South Africa	
	**None/incomplete primary**		26 (32.5)	0	6 (37.5)
	**Primary school**		53 (66.3)	3 (27.3)	10 (62.5)
	**Secondary school**		1 (1.3)	4 (36.4)	0
	**Higher education**		0	3 (27.3)	0

### Safety data reports by elicitation method

Of the 18 South African parent trial participants, 16 attended both study visits, of whom 15 (94%) reported differently between the general enquiry and checklists. Of 80 Tanzanian parent trial participants in the trial at the time of the nested study, 76 attended both study visits, of whom 65 (86%) reported differently between the general enquiry and checklists. For practical reasons the final sample size of those who participated in in-depth interviews were 11 in South Africa and 16 in Tanzania. Table 2 summarises the reports of adverse events, medical histories and concomitant medications elicited by question method.

**Table 2:** Summary of trial participants’ reports elicited by question method

	**South Africa**			**Tanzania**		
	**Medical histories**	**Adverse events**	**Treatments**	**Medical histories**	**Adverse events**	**Treatments**
Number of reports by general enquiry*	4	23	17	285	6	196
Additional number of reports by checklists (% change from general enquiry)*	8 (100.0)	20 (87.0)	23 (135.3)	245 (86.0)	1 (16.7)	2 (91.3)
Additional number of reports by interview **(% change from general enquiry and checklist)	4 (33.3)	1 (2.3)	4 (10.0)	15 (2.8)	0 (0)	9 (4.5)

*All participants in the sampling frame attending both visits (South Africa n = 16, Tanzania n = 76).

**Subset of participants who took part in in-depth interviews (South Africa n = 11, Tanzania n = 16).

There was an overall cumulative increase in number of reports from general enquiry, through checklists, to in-depth interviews with the largest increase in reports found in the checklists when compared with the general enquires. All additional AEs reported through use of the checklists or in-depth interview were rated as mild and unlikely to be related to the trial drug by trial staff; and no additional medical history or medications detected in using those methods changed eligibility for the trials. While there were large differences in the numbers of reports between the trial sites, it is not informative to make a direct comparison as, apart from the four-fold difference in sample sizes, the type of participant and trial designs were different. It is to be expected that otherwise healthy HIV-positive volunteers in South Africa have significantly fewer medical history reports compared to those in Tanzania who were presenting with multiple malaria symptoms; the South Africans could also report more AEs as they had a second opportunity to do so.

In the more detailed description of data reports (Additional file 2 and Additional file 3) it was observed that, in particular, baseline symptoms of nausea and vomiting in Tanzania appeared to be under-reported until they were specifically asked about. Similarly, body pain and fatigue were not reported until after specific questioning. However, this may be because they were captured within other terms (e.g. tingling or painful sensation, abdominal pain, chest pain, muscle pain and joint pain for body pain, and weakness for fatigue) in the Tanzanian questioning tool. Night sweats may have been reported within the concept of fever.

### Results from the trial participants’ narratives

**Figure 1:**
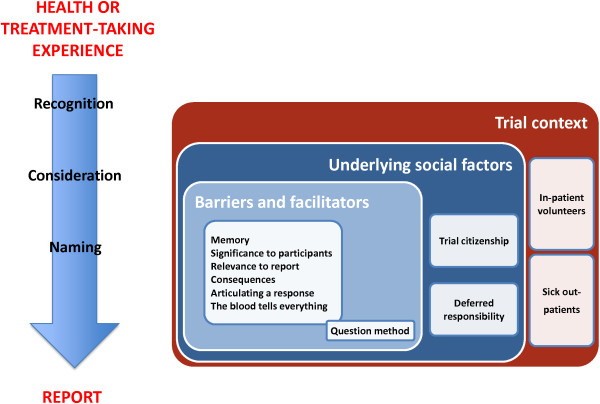
Diagram of trial participants’ narrative responses.

The qualitative data revealed that experiences of their health or use of treatments become data through a process including participants' recognising the information required, considering a reply, and effective articulation, or naming, of the response (Figure 1). The underlying social contexts could help to explain how specific barriers and facilitators to participant reporting manifest in these trial sites.

#### *Factors shaping participant reporting of health and treatments*

***Memory*** When discussing discrepancies between their responses to the different questioning (elicitation) methods, participants often said they forgot to report things in response to the general enquiry and, to a minimal extent, the checklist. Minor, intermittent and resolved health issues were more likely to be forgotten compared to those more severe, persistent or current. There were no consistent categories of treatments recalled more easily, except for ARVs, which could never be forgotten as they are considered so important to sustaining health:

*“I only used it [diclofenac, an anti-inflammatory] to reduce the tooth pain. When the pain stopped I [forgot] them. But with these ones [ARVs] I have no time to [forget] them, maybe God takes my spirit [fear of ill health/death from non-adherence]*” [In-depth interview (IDI) 11, Tanzania].

Participants predominantly attributed forgetting to report to the burden of having too much to remember, although two South Africans said it was due to significant memory decline since starting ARVs. The detail of the checklist, and to some extent the in-depth interview, was strongly viewed as helpful in overcoming memory problems.

**Significance to participants** When faced with a general enquiry, participants in both sites described conscious decisions to report current medical histories and AEs that were more bothersome, severe or persistent as opposed to those experienced as intermittent or less severe. Several South African participants also described decisions to first spontaneously report a new deterioration in health during the trial only when the symptoms worsened further. South African participants often recounted medical history in relation to when they initiated ARVs, when health was at a low point from AIDS-related illness or initial side-effects. Illnesses experienced since this period (major or minor) were commonly under-reported,

*“Ever since 2006 I don’t want to lie but I don’t feel anything… I always just get sick like ordinary people”* [IDI 4, South Africa].

This extended into reporting about AEs during the trial period, with declarations of good health followed by mention of health issues *“No, I was not sick at all. Just only coughing, flu, only that”* [IDI 8, South Africa].

Participant definitions or normalising of ill-health influenced when and how they revealed information in the in-depth interview. Categorising illness as 'major’ or 'bad’ was frequently associated with hospitalisation; tuberculosis treated as an outpatient was not being 'badly sick’, and only revealed in response to a specific question about tuberculosis. One participant did not mention her blindness in one eye until specifically asked.

Non-significance amongst Tanzanian participants sometimes reflected a slightly different concept. Rather than relating to classification of an experience as 'normal’ and therefore not reported, participants seemed to shape their perception of significance more around expectations of what would be significant to the doctor. When asked about symptoms by way of a general enquiry, those deemed the main problem(s) were chosen with expectation that others would be treated by default:

*I don’t think that you can tell the doctor one part after another that is in pain. … But you may decide to tell him the basic problem, or what is making me more sick. If I tell the doctor that I have fever he might give me the medicine … then all that I am feeling will calm down* [Focus group discussion (FGD) 1 Tanzania respondent 5].

Treatment use phraseology meanwhile revealed a hierarchy; after ARVs or antimalarials, use of intermittent or over the counter substances (such as painkillers or vitamins) were mentioned, but qualified with 'only’ or 'apart from’. These perceptions of significance may intersect with the next factor shaping reporting behaviour: relevance to report.

**Relevance to report** Participants appeared to delay reporting experiences that they perceived irrelevant, with the checklists helping them to decide what was necessary. Manifestation of relevance to report was, largely, different between sites. In South Africa, decisions to report were sometimes related to the trial’s objectives, i.e. whether the information would contribute to the success of the trial:

*“If I say after drinking the tablet I felt weak and tired … then they will write it … [as] the effects of the tablet. [To] them it gives …the wrong impression”* [IDI 5 South Africa].

History of sexual dysfunction, and poor memory (not on the checklists), were only revealed in the in-depth interview by two participants, who described these as irrelevant to the trial, and also untreatable, and therefore not useful to report. In Tanzania, relevance was chiefly attributed to the medical context. If a symptom at baseline was considered by the participant to be due to something other than malaria, such as work-related activities, it might not have been reported unless specifically prompted for. Similarly, if medication was bought for something other than malaria there was no reason to report:

*“I thought because I purchased it [pain killer] by myself because of the tooth pain I had no reason of telling him. But the Fansidar I purchased, I had a reason of telling him, because I can be tested and seen with malaria again. So they’ll understand, after the Fansidar being useless, what [they should] do*” [IDI 11 Tanzania].

**Consequences – attempts to control a situation** Participants in both sites described themselves or others in the trial withholding information in response to general questions and checklists for fear of negative consequences. These negative consequences were starkly different between sites. In South Africa, participants feared exclusion from the trial; there were three explicit, independent, second-hand reports of self-treatment with laxatives, non-adherence with ARVs, and gastro-intestinal illness that were withheld for this reason. When such scenarios were presented during the focus group discussion, participants denied that this happened, saying that misreporting could undermine the trial’s objectives. In contrast, Tanzanians considering consequences of reporting (or not) did not mention the trial; they were worried about going against hospital 'rules’. One said she did not report an anti-inflammatory taken during the trial for this reason. Most discussion on this topic in Tanzania, however, was in the focus group discussions and concerned fear of reporting traditional medicines:

*We who are living with [HIV], .. we are highly advised to refrain from traditional medicine issues because there is a difference between traditional medicines and these drugs. ….Yesterday I felt ill and went to dig muarobaini [neem tree root used for malaria] but without feeling any relief after drinking it. If I come to [doctor] today and he asks me what kind of medicine you used yesterday, telling him muarobaini….I must lie because already that is wrong. …That is due to fear of telling the doctor because we were already prohibited that thing. …There is some sort of [difficulty] telling the truth though it’s true, but the truth remains within me.* [FGD 1 Tanzania, respondent 3].

**Articulating a response** South African participants, who generally did not take long-term medications apart from ARVs, appeared to have a small personal formulary of named over-the-counter medications, but names of ad-hoc prescription medications were largely unknown. Though literate, one man displayed little interest in knowing what his prescription was. By contrast they had impressive detailed knowledge of their previous and current ARV regimens. In Tanzania, names of prescription medicines, including ARVs, were not known. As for HIV itself, ARVs were mentioned euphemistically, even when reverence appeared as high. In focus group discussions, Tanzanian participants initially cited their lack of education and an inability to read English labels, though it became clear that names of ARVs and other long-term prescription medicines are seldom verbalised during prescribing and dispensing. Meanwhile, equally complex names of antibiotics and antimalarials are known, because they are talked about in public.

Respondent: But I have not kept those [ARVs] in my mind. I have their container but I have never read it.

Interviewer: How come you knew amoxycillin?

Respondent: [laughs] They are simple to pronounce

Interviewer: How did you get that name?

Respondent: Because they are mentioned in the streets … like aspirin, simple names.

[IDI 4 Tanzania].

**The blood test tells doctors everything they need to know** When South African participants narrated what happened in the trial, this was largely about blood tests and examinations, with little mention of the verbal discussion with the clinician until they were prompted. While this could relate to the numerous blood samples taken in the trial ward and knowledge about the trial’s pharmacokinetic objectives, it persisted in their descriptions of follow-up visits, when tests were far fewer. When asked about the relative importance of blood tests versus being asked about their health and use of medicines, several declared the former as equivalent or superior.

“*[The test is] like they are asking about your health. … it’s the same thing*” [FGD 01 South Africa respondent 01].

Others said blood could reveal non-adherence to ARVs and use of any substance, including traditional medicines. Tanzanian participants did not display such a marked pattern, possibly because they expected to be asked about malaria symptoms and what antimalarials they had tried in order to inform treatment. However, when questioned about the importance of blood tests versus being asked about their health and use of medicines their discourse reflected that the former were considered a far superior source of information for the doctor. One participant indicated that questions were asked merely for the doctor to identify the more important test required.

#### Social context of the trials

While there may also be influence from psychological factors, conversations with participants in this study reveal social constructs that may underpin reporting behaviour. A minor construct “Being a subject” was common to both sites, though much stronger in South Africa; participants indicated they were beholden to certain trial-dictated behaviours, mostly regarding concomitant medication use. However, two more dominant constructs, “Trial citizenship” and “Deferred responsibility” may explain some of the differences observed between the sites.

**Trial citizenship in South African inpatient volunteers** South African participants described their important role, indeed sometimes their *work*, in facilitating the trial’s success. They were largely knowledgeable of, and aligned their work as trial participants with, researchers’ objectives. On hearing a participant was withdrawn due to a contra-indicated medication prescribed by her own doctor, they felt she was correct in reporting it even though she could not play her trial role anymore:

Interviewer: So, how did you feel about [another participant being withdrawn from the trial for taking a contra-indicated medication]?

Respondent 3: We feel bad

Respondent 5: We feel very bad

*Respondent 4: But [on] Saturday, the doctor told us [about] the side-effect [of] the tablets that the girl was taking for the skin. It will affect what? [Respondent 2: kidneys]. ... Then [that] girl, she can’t work with us*. [FGD 02 South Africa].

Even though participants conveyed this sense of responsibility towards the integrity of the trial, there were second-hand reports of others not playing their role properly, deliberately withholding information for fear of being withdrawn. Though loss of reimbursement was cited as a reason for this, one participant expressed it in relation to a worry about having to leave because participants had *“come [to] enjoy the trial”* [IDI South Africa, respondent 11]. This South African trial was conducted under stricter conditions than the trial in Tanzania in terms of, for example, admission for intensive pharmacokinetic sampling periods, and the foods and concomitant medications allowed. Being with each other and the staff for 72 hours offered a liberating experience; there was much mention of the opportunity to speak freely with others who understood what it meant to be HIV positive, phone numbers were swapped and focus group discussions were happy reunions. This may have nurtured the trial citizenship underpinning some decisions regarding relevance to report, which were sometimes made by consensus. Thus, for instance, an AE was considered unrelated to the South African trial if others were not experiencing it:

*“Somebody will wake up and say 'Guys I am feeling this, is anyone feeling it?’ And then because that one said no… you will also think 'Ah maybe it’s me. It’s only me*” [IDI South Africa, respondent 05].

Over and above these participants’ roles in this trial, however, there was recognition that they were monitored to avoid personal risk. Perhaps in addition to trial citizenship, the fact that the antimalarial trial drug was being used experimentally in participants without malaria meant that they had a heightened vigilance for possible side-effects:

*“I was busy trying to look for side-effects from the trial.*” [FGD 01 South Africa respondent 4] “*They asked me how did I feel.. and what the medicine [did] to me”* [FGD 02 South Africa, respondent 3].

**Deferred responsibility in sick Tanzanian outpatients** In Tanzania, particularly in the in-depth interviews, there was little discussion, or sometimes understanding, of the trial. Some participants showed how the locus of responsibility for knowing relevant information fell with trial staff as clinicians, rather than with themselves; the doctor had the knowledge to prompt them to reveal whatever additional information was required over and above what they respond to a general enquiry:

*“So I think the doctor understands more, that’s why he went on to probe. So I didn’t forget or wasn’t careless, but it’s my knowledge that is low, that if the stomach aches then even diarrhoea may be there, 'what about diarrhoea?*” [FGD 1 Tanzania, respondent 1].

Tanzanian participants were recruited to the trial individually when they presented to routine services with malaria symptoms for which they sought a cure. The trial was largely incidental to them achieving good health. There were few references to researchers’ priorities; it was personal:

*“I didn’t just join because of the name malaria, no. But it’s because there are tests and examinations that are being done on me so as to know how my health is. …So I was being researched. So I know how my state is, I was examined*” [FGD 1 Tanzania, respondent 1].

The experience predominantly described was of feeling ill, joining a project (to get optimal management), and thus feeling better. Participants considered the questions the doctor asked about health and treatments were for their personal benefit:

*“When he asked what kind of drug have you used, I suspect that he asked so that we don’t make it a habit buying drugs from drug shops, we should come and get tested first*” [FGD 1 Tanzania, respondent 4].

In the in-depth interview, responses to questions about change in health since baseline overwhelmingly concerned gratitude for improvement, malaria-related or not.

#### Trial clinicians' reflections on the limits of sensitivity

Participants in both sites had overwhelmingly recommended that more detailed questioning (checklists or in-depth interviews) helped them to report, and Tanzanian participants said that the focus group discussion taught them the importance of maintaining their own detailed illness and treatment record, for its personal health benefits. Trial clinicians, meanwhile, spoke of the challenge of eliciting comprehensive but relevant data when they will never know everything. For well-studied drugs, the focus of more detailed questioning could be on known or anticipated risks, combined with general enquires to detect anything else. But it was a quandary whether to probe for AEs that are perhaps insignificant or irrelevant to both clinicians and participants:

*“If they choose not to tell me about their headache when I’m asking them how they are and how they [feel] in their body…how severe could it be or… how important [is it] to them?“* [Trial clinician 2].

For trials of drugs with known safety profiles:

*“Isn’t the grading such a key issue? Because people’s lives are full of minor mishaps, and minor symptoms all the time. And if you really, really wring it out of people, you could generate a lot of irrelevant grade 1 stuff*” [Trial clinician 4].

There was, however, concern that selective detailed questioning could miss minor illness that impacts on adherence, and thus efficacy and an increased risk of malaria resistance at a population level. These clinicians, reflecting how patients may be intimidated by their role as doctors, despite them being at pains to be otherwise, suggested that other cadres of staff be involved in order to overcome barriers to reporting. This involvement, they said, could include designing elicitation strategies (social scientists), questioning participants (nurses or social scientists) or interpreting safety results (anthropologists).

## Discussion

Clinical trial guidelines have been developed to attain common standards across phases of drug development and disparate sites [28]. However, little is offered about methods for questioning participants to elicit information pertaining to harm and tolerability outcomes. The issue of whether to employ checklists in trials, as is routine in other pharmacoepidemiological study designs, and, if so, their components and mode of delivery, remains contentious. This study shows, as others have, that asking participants to indicate which of a checklist of items applies to them increases the sensitivity of detecting data compared to a general enquiry. In addition, some data were only detected during subsequent in-depth interviews, suggesting that checklists overcame some, but not all, factors involved in under-reporting in response to a general enquiry. In these two trials the additional data gained by more detailed or in-depth questioning did not alter eligibility for participation, or detection of AEs which staff determined to be related to the study drug(s). However, because of the inherent limitations of individual causality assessments, lost AEs may be ADRs when analysed statistically in a pooled analysis. The findings of this qualitative study suggest that contents of a checklist cannot feasibly be exhaustive enough to trigger all issues forgotten, deemed insignificant or irrelevant. It is also unlikely that checklists alone will overcome fears of reporting, by those who perceive themselves as either participants or patients.

### Data reports in context

This study offers empiricial evidence for the impact on antimalarial and ARV trial safety endpoints of individual perceptions of what is (and is not) memorable, significant, relevant, of personal consequence, articulable and necessary to report. Recall was a dominant factor, reflecting the focus on memory in the broader pharmacoepidemiological literature about validity of participant-reported data [11]. In addition, this study provides evidence of some less-studied influences on reporting, including participants’ perceptions of significance (whereby illness or treatment use may be normalised or gauged against another), relevance to report and negative consequences of reporting. Such factors influencing participant reporting in clinical trials are similar to those observed in other areas of epidemiology, suggesting that the evidence base for elicitation could be extended by learning from relevant methodological work conducted in other disciplines [29,30].

The study also identifies factors influencing elicitation processes and outcomes that could be more specific to the clinical trial context. Perceptions about relevance to report and fears of reporting appeared most likely to be influenced by differences between the trial sites. Others have explored how trial participants understand their identity, as being somewhere on a continuum from patient to active volunteer, and how this may shape outcomes [31-33]. This study describes how the South African participants appeared to take on a form of “trial citizenship”. Their prolonged opportunity to interact with staff in the ward is likely to have affected their understanding of the trial and what was important to researchers in a way that differed to the Tanzanian outpatient participants. The South African participants took on a responsible, job-like, role in determining the trial’s outcome. Beyond this, these otherwise healthy, HIV positive South Africans described their experience in the trial as a treat, a space to be free from a complicated life. The small community of participants in the trial ward, situated in a wider context of AIDS activism and social mobility, may reflect active biological citizens in allegiance with the conventional biomedical community of clinical trial staff [34]. The Tanzanian situation, meanwhile, is more likely to have represented a typical malaria therapeutic efficacy study whereby participants seek a cure from a clinic as usual and are focused on recovery. As observed elsewhere, despite consent information detailing experimental aspects of the trial, the consultations were understood by Tanzanians largely for their personal medical benefit [35].

### Locus of responsibility for assessing causality

This study did not reveal whether the design and contextual differences between the two sites fully explains the relatively high number of AEs in South Africa compared to Tanzania, though it may be that there was greater awareness of the focus on detecting side-effects in the former trial, compared with the latter where the focus appeared to be on resolution of malaria symptoms. South African participants were also relatively healthy, which may have increased their reporting of any deterioration in health. If so, this signals the potential for the trial design to influence how safety end points are understood and reported. While individual causality assessments made by staff during a trial allow for clinical decisions and adherence to regulatory reporting timelines, a central tenet is that evidence for causality is determined for registration purposes on the basis of aggregated data at the end of a trial or on meta-analysis with data from similar trials. The findings of this study suggest that implicit assessments of causality by participants can occur very early in the process of data generation, undermining the assumptions behind the 'objective data’ used to determine harm and tolerability. Participants make decisions to report based on their personal logical assessments of causality, severity and risk-benefit. Thus, the lines between adverse events and suspected adverse drug reactions according to ICH definitions of causality are blurred at the point of questioning [28].

### The importance of treatment literacy

There were significant differences in how ARVs were talked about by participants in these two sites. Treatment literacy - i.e. knowing about medications one is taking - is fundamental to pharmacoepidemiology, especially where record linkage is difficult. Yet appropriate ways of eliciting concomitant medication reports in trials is a particularly neglected research area. Lay public definitions of what constitutes a medication may explain observed delays in reporting some medications versus others [36], and this study’s data from Tanzania suggest that drugs considered not relevant to the hospital are not reported. Others have shown how people may not know names of prescribed medications, so rather leave this knowledge to a health practitioner [37]. This behaviour may be more likely in more paternalistic healthcare systems [38]. However, HIV is a particularly stigmatised condition, and references to it (and its treatment) may also be subtle, even by providers. While South Africans also appeared not to know names of prescription-only drugs, their ability to intimately know ARVs could be a result of greater progress in de-stigmatising HIV/AIDS, particularly in a setting where participants are likely to be empowered by well-known advocacy groups and treatment programs [39]. Though the Tanzanian respondents did not express themselves as trial citizens, their usual experience of malaria management was certainly enhanced by the specialised, dedicated trial team. By the time of their focus group discussions they spoke more knowledgeably about the trial, expressed satisfaction with the focus group discussion allowing them to socialise, dropped euphemisms for HIV, and proposed they take more responsibility for keeping health and treatment records. This suggests there is scope for altering what could be the natural course of a patient-health facility relationship to overcome specific impediments to trial reporting. However, this would need careful consideration in view of evidence that moving away from usual care to the trial context may influence how information that becomes safety end points are understood and reported [40].

### Blood tests versus reports

Blood taking is a strong symbol of biomedicine, and there may be unrealistic expectations about the capabilities of blood tests in trials [35]. The privileging of blood over recounted illness and treatment experiences may reflect the recognition of a technological imperative in biomedicine; that tests and equipment have the power to provide doctors with the answers they require [41]. This may be reinforced by a lack of opportunity to describe illnesses, with health workers indicating what is important to say, hear and know [42]. If trial participants or patients are to be encouraged to relate their experiences of illnesses and concomitant medications, they may need reassurance that their experience is of equal importance and that blood tests may not provide the same information. Those eliciting responses may need to show particular patience in listening to stories that surround trial data end points.

### Limitations

This study does not reveal a whole 'truth’, as participants’ recognition of, and willingness to report information vary, and are subject to the trial clinicians' and researchers’ interpretations. Other than lack of a gold standard, a potential limitation is that participants who did not report differently between general enquiries and checklists were systematically different from those who did. As the goal was to explore reasons for non-reporting, this study focused on the latter, but future work could include the former to understand reporting more generally. Interviewing participants immediately after each visit where general and checklist enquiries were compared could have increased the validity of comparisons, but could also have led to participant fatigue. Thus, it was opted to use time and budget judiciously, delaying in-depth interviews until after Day 7. The sample size was too small to detect statistically significant differences in responses to the 3 questioning methods and it was not possible to measure influence from other factors, such as the characteristics of the trial clinicians and interviewers (including role and gender), precise mode of delivery of questions, or influence from the participant trial information. The questioning methods were compared in a sequential design rather than in a direct parallel manner as this was a realistic representation of how these methods are generally used in trials. The study results may not be generalizable to other trials where the methods to detect safety data and context are very different.

## Conclusion

This study offers more evidence that questioning methods influence the detection of clinical trial safety data. This could prove a major limitation to optimal safety assessments, and preclude valid pooled data safety analyses. The study offers explanations for this phenomenon through the voices of participants, indicating that different trial contexts cultivated some specific conditions that had a role in mediating recognition, reporting and articulation of important variables. Based on this work, it makes sense to develop appropriate messages for participants to convey the concept that checklist items, if used, are merely examples of the level of data required, and that their answers to questions about ill health and treatment use are as important as tests and examinations. This may particularly help those believing it is the health worker alone who assumes responsibility for reports. Participants could also be counseled to quell potential fears about reporting and appropriate ways to manage prohibited concomitant medications reports without 'punishment’ could be explored. For any elicitation method, conceptualisations of the purposes of elicitation, including those that trial staff hold, should be incorporated in its design to help negate potential areas of mismatch. There is a need to find optimal phraseology to ensure understanding of question terminology. In areas with low treatment literacy, pictures or samples could help staff uncover the names of concomitant medications. Pictorial methods may also be of value in enhancing communication, including the rationale for the information needed [43]. The challenge in incorporating these in clinical trials is the need to be systematic whilst being locally interpretable. Despite their limitations, checklists are an obvious focus, a way forward being to explore, over and above content, their mode of application. It is here that consideration of possible influences from a trial’s social context would be most useful. Clinical researchers could also consider what role their participants may take on, for example that of a patient or a trial citizen. Further methodological work should be embedded in clinical trials to investigate influences on the measurement of participant-reported endpoints [44]. Understanding contextual factors influencing trial outcomes could form the basis of innovative ways to capture important safety data outcomes [45].

## Competing interests

The authors declare that they have no competing interests.

## Authors’ contributions

EA conceived of the study, led design, data collection and interpretation, conducted South African interviews and wrote the paper. CC had significant input throughout all these stages. AM and IM conducted Tanzanian interviews. AM refined study tools and supervised the qualitative team in Tanzania. KB, SS and UM contributed to study design and data interpretation. ML and LV supervised the Tanzanian team, LV also contributed to data interpretation. All authors contributed to and approved the final paper.

## Pre-publication history

The pre-publication history for this paper can be accessed here:

http://www.biomedcentral.com/1471-2288/13/140/prepub

## Supplementary Material

Additional file 1 Description of data: checklist items by body systems, symptoms, diseases and treatments. Click here for file

Additional file 2 Description of data: detailed description of South African participants’ reports of adverse events, medical histories and concomitant medications elicited by question method. Click here for file

Additional file 3 Description of data: detailed description of Tanzanian participants’ reports of adverse events, medical histories and concomitant medications elicited by question method. Click here for file
